# Attitudes towards domestic violence in 49 low- and middle-income countries: A gendered analysis of prevalence and country-level correlates

**DOI:** 10.1371/journal.pone.0206101

**Published:** 2018-10-31

**Authors:** LynnMarie Sardinha, Héctor E. Nájera Catalán

**Affiliations:** School for Policy Studies, University of Bristol, Bristol, United Kingdom; The University of Warwick, UNITED KINGDOM

## Abstract

**Background:**

Violence against women by an intimate partner (DV) is a serious public health and human rights issue. Attitudes justifying DV strongly predict its perpetration and victimisation. This paper presents gendered ecological analyses of the societal acceptance of DV in 49 low- and middle-income countries (LMICs) across geographical regions.

**Methods and findings:**

We utilised data from 49 Demographic and Health Surveys conducted between 2005 and 2017, United Nations Statistics and topic-specific meta-databases. DV acceptance was measured as the justification of ‘wife-beating’ in at least one of five circumstances, and by the summative scale. Stepwise multiple linear regression examined country-level social, economic and political empowerment predictors of societal acceptance of DV amongst women, men, and the aggregate gender difference. Women were more likely than men to justify DV in Sub-Saharan Africa and South (east) Asia with societal acceptance of DV being more widespread in these regions compared with Latin America, the Caribbean, Central/West Asia and Europe. Political conflict and limited economic rights for women were associated with higher levels of DV acceptance amongst women and men. Men in more democratic countries were less likely to justify DV. Amongst women, higher national female literacy rates predicted lower levels of justification. There were higher levels of DV acceptance amongst women and a wider aggregate gender difference in countries with a larger representation of women in national parliament.

**Conclusion:**

Justification of DV is widespread amongst women and men in LMICs with acceptance rates varying across countries and regions. Gender differences in the impact of contextual factors on DV acceptance supports a gendered approach to national-level interventions. Our findings highlight the need for tailored interventions targeting DV acceptance in conflict-impacted societies. The emphasis of inter(national) policies on the ‘empowerment’ domains of widely-used gender (in)equality indices need to be coupled with strategies tackling discriminatory gender norms.

## Introduction

Societal attitudes to domestic violence against women by an intimate partner (DV) play a key role in the perpetration of DV, women’s responses to their victimisation, disclosure and help-seeking behaviour, and the wider community’s and society’s responses to DV [1]. Over 35 population-based studies from Asia, Africa and the Middle East have demonstrated that attitudes condoning DV and discriminatory gender norms around male authority and control are highly predictive of rates of perpetration and victimisation (for example [1–13]). Given that one in three women globally have experienced physical/sexual violence from an intimate partner at least once in their lifetime [14, 15], DV is the most common form of violence against women. It is a human rights violation and has been recognised as a public health issue of epidemic proportions with negative short- and long-term impacts on women’s and children’s physical and psychological health and well-being. It has socio-economic consequences for families, and more widely impacts on the social and economic development of countries [16]. Primary prevention of DV is both urgent and vital, however, much remains to be accomplished in the evidence-based prevention, not least in better understanding societal attitudes to DV that have been identified as a central part of the “social ecology” of violence against women [17].

There is a growing body of research, including some multi-country studies, on the structure of attitudes to DV at the individual or household level in low-and middle-income countries [2–12, 18–21]. However, there is little cross-national country-level evidence [22, 23] on how social, economic and political empowerment strategies, accepted as indicators of gender (in)equality influence the societal acceptability of DV. At the macro-level, Heise’s social ecological framework references gender norms including the acceptance of DV, systems that privilege men’s entitlement, rigid gender roles, masculinities linked to aggression and dominance, and honour as important drivers of violence against women [17, 24, 25]. A fundamental premise of this study is that DV is a manifestation of unequal power between men and women. The acceptance of unequal gender roles and justification of DV in a society is seen as indicative of the status of women. Hence the theory that policies and programmes to improve the status of women and facilitate their participation at all levels of development will reduce DV is reasonable and compelling. However, to date, there is limited empirical data to support it [17, 26–27].

Discriminatory formal and informal social institutions [28–30] including gender norms and the acceptance of DV are deeply rooted in and cannot be separated from their geographical, socio-cultural, economic and political settings. Building on a nascent evidence-base, this multi-country study addresses an important knowledge gap on how different systemic economic, social and political empowerment factors including those related to wider gender inequalities such as national female literacy rates, multidimensional poverty, women’s labour force participation, women’s participation in public life, the existence and quality of laws on DV, political conflict and levels of democracy influence societal attitudes towards DV amongst women and men across national and geographic settings.

Furthermore, examining the gendered pattern in societal attitudes towards DV and the gender differences in the contextual drivers of these attitudes is an important step towards implementing appropriate gender-sensitive national- and international-level DV prevention policies and intervention programmes. However, few population-based multi-country studies have adopted gender-centric analyses of men’s and women’s attitudes to DV in low- and middle-income settings [18–21, 23].

In this paper we examine the prevalence and pattern of attitudes towards DV amongst women and men across 49 low- and middle-income countries, the country-level economic, social and political predictors of its societal acceptance and how the impact of these factors on attitudes may differ for women and men.

## Methods

### Data and sample

This paper draws on several sources of data including 49 Demographic and Health Surveys (DHS) conducted between 2006 and 2017 and available as of February 2017. The DHS (sometimes referred to as the National Family Health Survey or the Maternal and Child Welfare Surveys, for example, in different countries) are nationally representative, cross-sectional, household sample surveys with large sample sizes and robust multi-stage cluster sampling techniques. As a gendered analysis was central to this study only the 49 low- and middle-income countries with comparable DHS data on women’s and men’s attitudes to DV and measures of women’s empowerment are included in our sample. The most recent survey per country was used. The 49 country surveys represent 1,174,108 respondents that included women aged 15–49 years and men aged 15–59 years. (S1A Table). The survey year matched prevalence rates of DV experienced by women in the sampled countries are also presented in the Appendices (See S1B Table).

Country-level metadata were accessed from different UN Statistics, multilateral and topic specific databases to construct a pooled database. All data were harmonised prior to analyses.

### Ethical considerations

The DHS has especially trained interviewers to administer the survey. Consent was obtained from all respondents. The interviews were conducted to maximise privacy and safety. All identifier information is removed. The surveys were approved by the Ethics Committee of the ICF International and by the National Ethics Committee in each of the countries. (See https://dhsprogram.com/What-We-Do/Protecting-the-Privacy-of-DHS-Survey-Respondents.cfm for details). This study was approved by the Ethics Committee of the University of Bristol.

### Outcome variable

The DHS measured attitudes towards DV using 5 items. Respondents were asked if a husband is justified in hitting or beating his wife if: “she burns the food,” “she argues with the husband,” “she goes out without informing the husband,” “she neglects the children,” and “she refuses to have sex with the husband". Response options were ‘Yes’, ‘No’ and ‘Don’t know’. Four sets of country-level outcome variables were created by aggregating the individual-level data as follows: two binary outcome variables for women’s and men’s acceptance rates of wife-beating coded as '0' if the response to any one of the five scenarios was ‘no’ and ‘1’ if they responded ‘yes’. When aggregated across individuals, attitudinal measures can serve as a reasonable proxy for norms that prevail in a setting and measure the prevalence rate of societal acceptance of DV (13). Using the first set of country-level outcome variables the survey-level variable of the ‘gender difference in acceptance of DV’ was computed (difference in the proportion of women justifying DV and men justifying DV in at least one situation). An ‘Attitudes to DV’ additive scale ranging from 0 to 5 was constructed after conducting reliability analyses per country and of the pooled data (Cronbach’s alpha = 0.86). This is a country-level mean score where higher values on the scale reflect higher levels of justification of DV and vice versa.

### Explanatory variables

The country-level social, economic and political empowerment measures and data sources are thematically summarised in S2 Table. For each of the country-level predictors, data matching the DHS survey year was used, and where this was unavailable, data for the closest year preceding the survey date was used. This global scale study examines a comprehensive range of risk and protective factors associated with the acceptance of DV rather than, for example, the use of less nuanced composite indices like the Human Development Index or the Gender Inequality Index. A robust household-level multidimensional deprivation index was constructed in line with the Sustainable Development Goals using the DHS data on housing materials, access to basic healthcare, sanitation, safe drinking water and primary education. A mean country score was computed, i.e. the higher the value the more severe the material deprivation. This index has advantages over the DHS Wealth Index which is a relative index constructed for each country at the time of the survey and is useful for studying within country equity and relative economic poverty. With each having a mean value of zero and a standard deviation of one specific scores cannot be directly compared across countries or over time.

### Statistical analyses

Prevalence estimates of societal acceptance of DV are presented as country-level percentages of women’s and men’s justification of DV in at least one circumstance. Spearman’s rank correlation examined the unadjusted relationship between the contextual-level factors and societal levels of DV acceptance. Multivariate analyses using stepwise linear regression models identified the independent influence of significant economic, social, and political indicators of women’s and men’s acceptance of DV at the country-level. Data were screened for multivariate outliers and none of the cases had an undue influence on the model (Cook’s distance<1). The scatterplot and histogram of the distribution of residuals along with the normal probability plot confirmed that model assumptions (normality and homoscedasticity of residuals) had been met. Collinearity diagnostics indicated no evidence of multicollinearity. A total of 18 models were estimated where all outcome variables (but the gender gap) are skewed and zero-bounded (censored). Tobit and linear regression models were fitted to assess the effect of truncation upon the estimates. Both model strategies yielded the same results and therefore only the coefficients from the linear regression models are presented. Stata v14 was used to conduct all analyses.

## Results

### Prevalence and distribution of women’s and men’s acceptance of DV

The prevalence of DV-supportive attitudes ranked within- and between-regions is presented in Table 1. Societal acceptance of DV was lowest in the Dominican Republic (3.13%) and was most widespread in Timor-Leste (83.47%). Over one in three people (36.40%) across these 49 countries justified DV in at least one circumstance. Regional averages suggest that women and men in Latin America and the Caribbean (11.87%), and Central/West Asia and Europe (28.55%) have less widespread violence-justifying attitudes compared to their counter-parts in Sub-Saharan Africa (38.40%) and South/South-East Asia (46.78%). There is, nonetheless, a large degree of variation across countries and within global regions with especially high rates of DV acceptance in conflict-affected countries. For example, within Sub-Saharan Africa whilst 12.58% of the population in Malawi justified DV, its acceptance in the Democratic Republic of Congo was close to 70% and 79.12% in Guinea. Similarly, in the South and South -East Asia region Maldives had the lowest prevalence rates of DV-justifying attitudes at 13.79% but within the same region Timor-Leste and Afghanistan (76%) had the highest prevalence of pro-DV attitudes amongst the 49 sampled countries.

**Table 1:** Prevalence of attitudes justifying domestic violence in the 49 low- and middle-income countries across geographical regions (weighted).

Region/Country	Proportion of women and men who ever justified DV			Mean Score: ‘Attitudes to DV Scale’ (Scale range = 0–5)		
	Women	Men	Both	Women	Men	Combined
*Latin America & the Caribbean*						
Dominican Rep	2.35	3.90	3.13	0.04	0.07	0.06
Honduras	12.36	9.39	10.88	0.25	0.19	0.22
Haiti	16.74	14.65	15.70	0.33	0.28	0.31
Guyana	16.29	19.26	17.78	0.32	0.35	0.34
**Region Average**	**11.94**	**11.80**	**11.87**	**0.24**	**0.22**	**0.23**
*Central/West Asia and Europe*						
Ukraine	3.56	11.12	7.34	0.05	0.21	0.13
Armenia	9.30	19.93	14.62	0.17	0.41	0.29
Moldova	20.81	21.7	21.26	0.38	0.41	0.40
Albania	29.75	36.35	33.05	0.66	0.81	0.74
Kyrgz Republic	33.71	50.36	42.04	0.79	1.15	0.97
Azerbaijan	48.98	56.95	52.97	1.35	1.41	1.38
**Region Average**	**24.35**	**32.74**	**28.55**	**0.57**	**0.73**	**0.65**
*Sub-Saharan and North Africa*						
Malawi	12.57	12.63	12.58	0.29	0.26	0.28
Benin	16.22	14.85	15.54	0.40	0.30	0.35
Mozambique	21.00	18.76	19.88	0.41	0.30	0.36
Ghana	28.26	12.49	20.38	0.73	0.28	0.51
SaoTao Principe	19.51	21.37	20.44	0.41	0.47	0.44
Togo	28.74	17.59	23.17	0.77	0.41	0.59
Namibia	28.23	21.26	24.75	0.62	0.39	0.51
Swaziland	23.17	31.27	27.22	0.44	0.61	0.53
Comoros	38.96	16.24	27.60	1.06	0.31	0.69
Kenya	19.67	36.22	27.95	0.46	0.81	0.64
Nigeria	34.74	24.66	29.70	1.04	0.59	0.82
Madagascar	32.30	29.11	30.71	0.70	0.64	0.67
Liberia	42.49	24.23	33.36	1.12	0.53	0.83
Lesotho	33.32	38.79	36.06	0.73	0.82	0.78
Zimbabwe	39.55	33.04	36.30	0.84	0.63	0.74
Burkina Faso	43.46	33.39	38.43	1.21	0.74	0.98
Zambia	46.86	30.73	38.8	1.49	0.71	1.10
Rwanda	56.21	24.74	40.48	1.68	0.54	1.11
Senegal	57.18	26.76	41.97	1.94	0.68	1.31
Cameroon	46.46	37.94	42.20	1.12	0.83	0.98
Niger	59.59	26.00	42.80	2.20	0.71	1.46
Cote d’Ivoire	47.88	40.97	44.43	1.31	0.99	1.15
Gabon	50.24	38.61	44.43	1.05	0.74	0.90
Gambia	58.40	32.28	45.34	1.57	0.75	1.16
Sierra Leone	62.79	33.54	48.17	1.97	0.80	1.39
Tanzania	58.05	39.63	48.84	1.81	0.98	1.40
Uganda	58.28	42.75	50.52	1.50	0.98	1.24
Ethiopia	68.43	44.68	56.56	2.26	1.25	1.76
Burundi	72.93	43.37	58.15	2.13	0.96	1.55
Mali	76.35	52.81	64.58	2.47	1.42	1.95
Congo Dem Rep	74.76	59.53	67.15	2.26	1.49	1.88
Guinea	92.06	66.17	79.12	3.57	1.95	2.76
**Region Average**	**44.95**	**31.85**	**38.40**	**1.29**	**0.74**	**1.02**
*South & South East Asia*						
Maldives	30.77	13.79	22.28	0.74	0.30	0.52
Indonesia	34.48	17.31	25.9	0.68	0.31	0.5
Pakistan	42.23	31.91	37.07	1.43	0.78	1.11
Cambodia	49.82	26.07	37.95	1.23	0.49	0.86
India	47.23	41.77	44.50	1.29	0.98	1.14
Afghanistan	80.21	72.39	76.30	2.26	1.61	1.94
Timor-Leste	86.18	80.75	83.47	2.85	2.44	2.65
**Region Average**	**52.99**	**40.57**	**46.78**	**1.50**	**0.99**	**1.25**
**49 country average (min/max)**	**41.09**	**31.71**	**36.40**	**1.15 (0.04/3.57)**	**0.74 (0.07/2.44)**	**0.95 (0.04/3.57)**

The five attitudinal items were combined additively to construct the ‘Attitudes to DV’ scale ranging from 0–5. The last three columns in Table 1 suggest a strong positive association between the severity of the justification of DV (as measured by the mean scores) and the prevalence of DV-supportive attitudes: countries and geographical regions where women and men have a higher mean score on the justification of DV scale were also the countries and regions with more widespread societal acceptance of DV. For example, in the Dominican Republic with the lowest prevalence of societal acceptance of DV also had the lowest mean score (M = 0.06) compared with Timor-Leste (M = 2.65) or Guniea (M = 2.76) where the prevalence of violence-justifying attitudes was very high. The levels of DV justification was highest in South (east) Asia (M = 1.25) and Sub-Saharan Africa (M = 1.02) as compared to Latin America and the Caribbean (M = 0.23) and Central/West Asia and Europe (M = 0.65).

**Fig 1:**
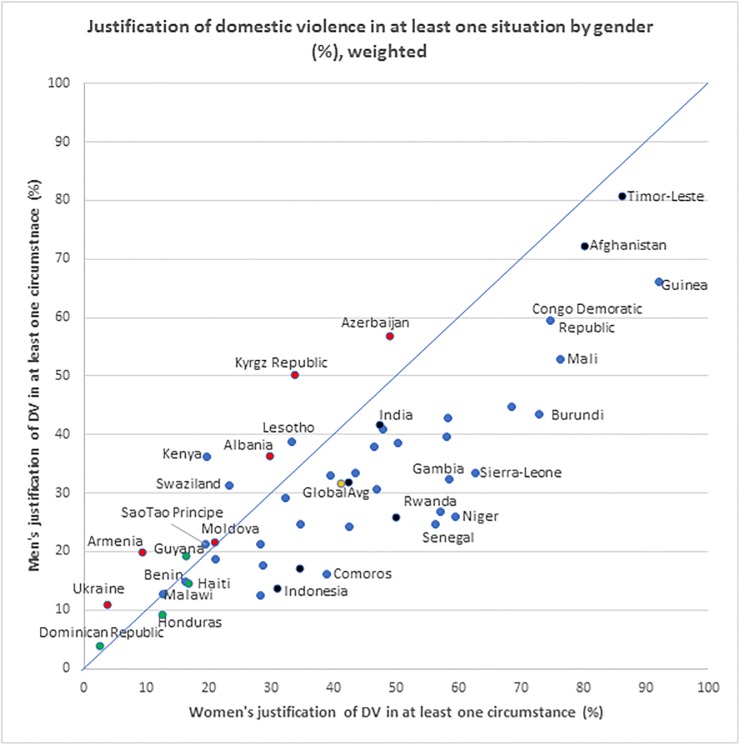
Gender differences in the justification of domestic violence across the 49 low- and middle-income countries (weighted).

Fig 1 visually presents the gender disaggregated population prevalence of DV justification across the 49 countries. Each data point represents the proportion of women and men justifying DV in at least one of the presented circumstances with the 45 degrees line representing the line of equality between men’s and women’s attitudes. A larger proportion of women justified DV in a majority of countries (36 of the 49) including all the South (east) Asian countries, and in 33 of the 37 countries in Sub-Saharan Africa with the exceptions of Kenya, Lesotho, Swaziland and Malawi. On the other hand, in most of the countries in Central West Asia and Europe in Latin America and the Caribbean a larger proportion of men justified DV. Across the 49 countries 41% of women and 32% of men justified DV.

**Fig 2:**
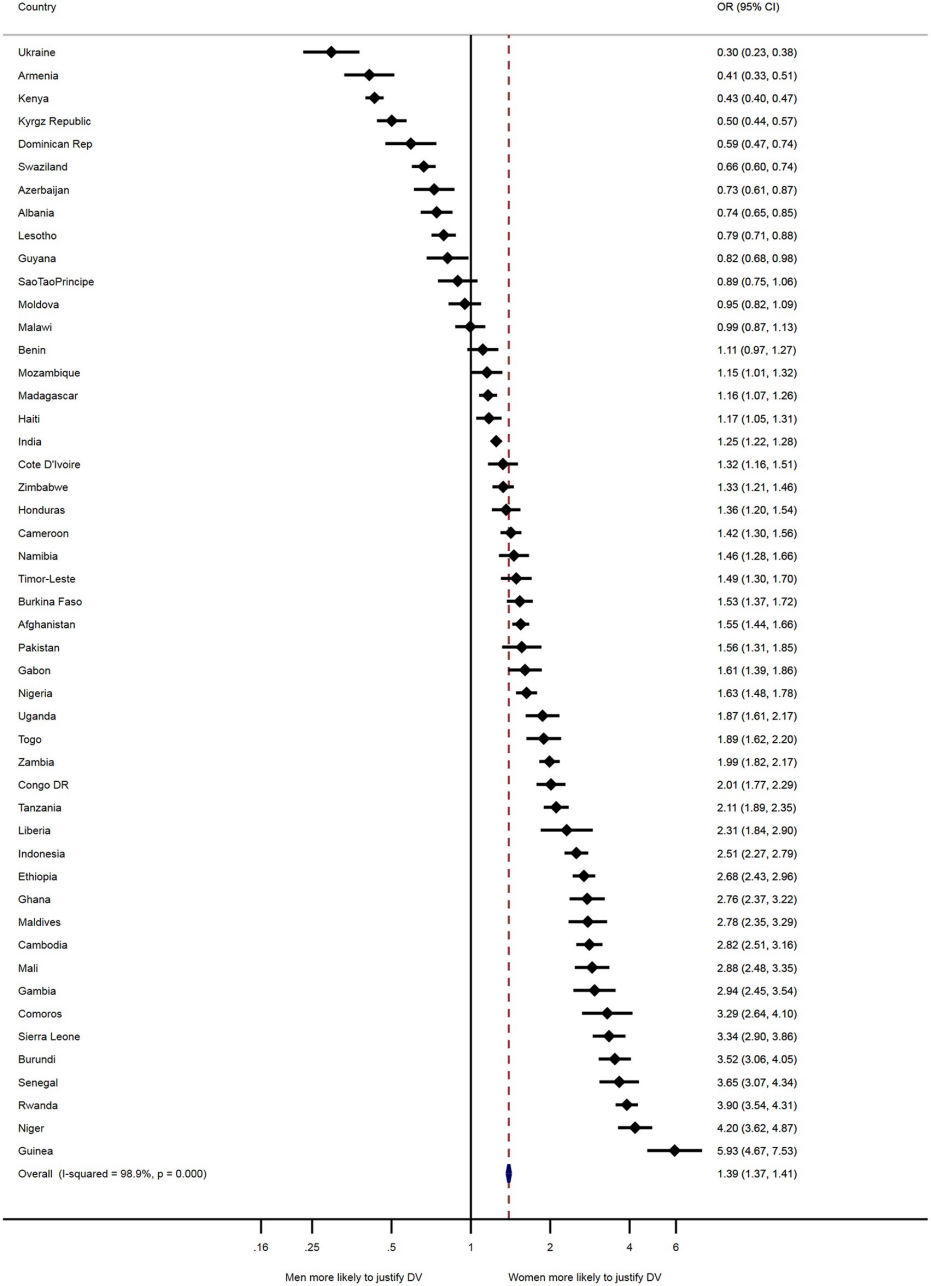
Forest plot of weighted gender-difference in attitude towards domestic violence of 49 low- and middle-income countries.

Extending Uthman, Lawoko and Moradi’s descriptive metanalysis technique [21], Fig 2 presents the odds ratios (OR) and 95% confidence intervals (CI) from individual countries and the pooled result. The calculated pooled effect estimate assuming a fixed-effects model was OR = 1.39; 95% CI 1.37–1.41. The Cochran’s Q test (Q = 4299.34; *p* < .001) and the corresponding *I*^2^ (99%) indicated statistically significant heterogeneity. In the leave-one-country-out sensitivity analysis the CIs did not change materially with exclusion of any of the countries, which remains within 95% confidence interval of the overall estimate for all countries. This analysis confirmed the stability of the results.

Fig 2 suggests that most countries with high levels of DV justification also had substantially wider gaps in women’s and men’s acceptance of DV. Furthermore, in these countries with higher societal acceptance of DV, women were more likely to justify DV compared with men. For example, the largest gender differences were in Guinea, with women being almost 6 times more likely than men to justify DV (OR = 5.93; CI 4.67, 7.53). In Niger (OR = 4.30; CI 3.62, 4.87) and Rwanda (OR = 3.90; CI 3.54, 4.31), women were 4 times more likely than their male counterparts to justify DV. However, Fig 2 also indicates that even in some countries with lower prevalence of societal acceptance of DV there were substantive gender gaps such as in Ukraine where men were over three times more likely to justify DV than women (OR = .30; CI .23, .38) and in Armenia men were almost twice as likely to justify DV (OR = .41; CI = .33, .51). Gender differences were not statistically significant in Malawi (OR = .99; CI .87, 1.13), Moldova (OR = .95; CI = .82, 1.09) and Sao Tao Principe (OR = .89; CI .75, 1.06) and in Benin (OR = 1.11; CI .87 1.13). As indicated by the overall pooled result, globally women were 1.39 times more likely than men to justify DV (OR = 1.39; CI 1.37, 1.41).

### Contextual-level determinants of societal attitudes to domestic violence

#### Results from bivariate analyses

Bivariate analyses indicate that amongst the social and economic correlates lower levels of multi-dimensional deprivation, higher national female literacy rates and greater economic rights for women were associated with lower levels of societal acceptance of DV amongst women and men (Table 2). National rates of female secondary and tertiary education, and prevalence of early marriage in a country were significantly correlated with lower acceptance of DV only amongst women.

**Table 2:** Bivariate associations of contextual indicators and women’s and men’s acceptance of domestic violence in at least one situation (N = 49).

	Women	Men	Gender Difference
**ECONOMIC AND SOCIAL FACTORS**			
Gini Inequality Index	(-0.26)	-0.35*	(0.01)
Multi-dimensional Deprivation Index	0.53***	0.34**	0.44**
Female literacy rates	-0.59***	-0.31*	-0.59***
Female primary education	(-0.14)	(-0.15)	(0.02)
Female secondary education	-0.42*	(-0.21)	-0.47**
Female tertiary education	-0.42**	-0.30*	-0.39**
Female labour force participation	(-0.05)	[-0.16]	(0.16)
Women working for cash	(-0.09)	(-0.08)	(-0.09)
Women’s economic rights	-0.49**	-0.47**	-0.31*
Early Marriage (<18)	0.33*	[0.25]	0.30*
**POLITICAL FACTORS**			
Women in national parliament	(0.24)	(0.22)	(0.20)
Women’s political rights	(0.18)	(0.21)	(0.13)
Unified democracy mean score	-0.42**	-0.41**	(-0.24)
Conflict mean (average 5 year score)	0.46**	0.40**	0.32*
**LEGISLATION**			
Existence of explicit DV law	-0.14	-0.25	(0.10)
Overall quality of DV law	0.25	0.21	(0.25)
CONTROLS			
GDP PPP per capita	-0.36**	-0.11	-0.46***

NOTE: The outcome variable for women and men was computed as the percentage of responses marked as ‘Yes’ to any one of the given statements. The difference between the proportion of women and proportion of men justifying DV in at least one circumstance is the outcome variable for the gender difference. () indicates a non-significant Spearman’s Rho test statistic at the 0.5 level.

*p < .05;

** < .01;

***p < .001

Higher levels of democracy and lower levels of political conflict were significantly correlated with lower levels of DV acceptance at the national level. The existence of an explicit law against DV and the quality of the law, however, were not significantly associated with national levels of DV acceptance.

Levels of female education, poverty, prevalence of early marriage and women’s economic rights were correlated with the gender gap in the societal acceptance of DV (Table 2). Among the political and legislative factors, only conflict within the past 5 years was significantly associated with the gender difference in attitudes to DV.

#### Results from multivariate analysis

Table 3 presents the results of the linear regression analyses of the country-level economic, social and political predictors of women’s and men’s acceptance of DV (at the aggregate level). The regression models first examined the economic and social predictors of women’s and men’s attitudes to DV and then, separately, the political factors (S3A–S3D Table) before constructing the combined model.

**Table 3:** National-level economic, social and political predictors of women’s and men’s acceptance of domestic violence: Multiple linear regression.

	Women		Men	
	Model 1	Model 2	Model 3	Model 4
**Multidimensional deprivation score**	.202* .019 .386	-.072 -.290 .146	-.008 -.113 .098	-.085 -.227 .058
**Inequality Index (Gini)**	-.013 (-.029 .003)	-.009 (-.023 .005)	-.008 (-.017 .001)	-.007 (-.016 .002)
**Women’s economic rights**	-.351* (-.657 -.048)	-.335** (-.597 -.074)	-.177* (-.352 -.002)	-.173* (-.344 -.001)
**Existence and quality of DV law**	.042 (-.041 .124)	.016 (-.056 .088)	.021 (-.026 -.068)	.013 (-.034 .061)
**Democracy Score**	-.147 (-.602 .308)	-.275 (-.671 .122)	-.262* (-.0523 -.001)	-.298* (-.558 -.038)
**Women’s political rights**	-.645 (-1.390 .099)	-.406 (-1.058 .246)	-.169 (-.596 .258)	-.102 (-.529 .325)
**Seats held by women in national parliament**	.028* (.007 .048)	.025** (.009 .194)	.007 (-.005 .018)	.006 (-.006 .018)
**Political conflict**	.069 (-.025 .190)	.101** (.008 .043)	.073* (.011 .134)	.078* (.017 .139)
**Female literacy rates**	-	-1.760*** (-2.727 -.793)	-	-.495 (-1.129 .139)
**BIC used by Stata**	99.199	88.113	50.277	50.920
**Adjusted R squared**	.456	.600	.385	.411

NOTE: The Attitudes to DV scale is the outcome variable; The unstandardised beta coefficients and the 95% CI are presented.

***p < .001

** p < .01

*p < .05;

Based on significance and model fit female primary, secondary and tertiary education, and female labour force participation were not included in final model.

#### Multivariate predictors of women’s and men’s acceptance domestic violence

As indicated by Table 3 greater women’s economic rights was a predictor of lower levels of DV acceptance by both women and men at the country level with a larger effect for women (B = -.335, p < .05) than for men (B = -.173, p < .05). Whilst the societal acceptance of DV, especially amongst women, was higher in poorer countries (B = .202, p < .05) this was no longer significant once the country’s female literacy rate was introduced into the model. Higher levels of female literacy was the strongest protective factor for women’s societal acceptance of DV (B = 1.760, p < .001).

Political conflict was a significant risk factor for higher levels of DV acceptance amongst both women (B = .025, p < .05) and men (B = .078, p < .05). This effect of conflict on men’s acceptance of DV was stronger than that for women. There was lower acceptance of DV amongst men in more democratic nations (B = .298, p < .05). Contrary to expectations, a greater representation of women in national parliament was associated with higher levels of DV acceptance amongst women (B = .101, p < .05).

#### Multivariate predictors of the gender difference in acceptance of DV

Table 4 presents the national social, economic and political predictors of the aggregate gender difference in the acceptance of DV. As with the previous models, a country’s multidimensional deprivation score significantly predicted the gender gap in DV attitudes (B = 5.735, p < .01) before accounting for national female literacy rates. National female literacy rates emerged as the strongest predictor of aggregate gender difference in DV acceptance (B = -.283, p < .01). Women’s representation in national parliament and the existence of national legislation on DV also significantly predicted larger gender gaps in the societal acceptance of DV.

**Table 4:** Socio-economic and political predictors of the gender difference in attitudes justifying domestic violence: Multiple linear regression.

	Model 5	Model 6
**Multi-dimensional deprivation score**	5.735** (1.99 9.478)	1.330 (-3.446 6.105)
**Inequality index (Gini)**	-.061 (-.388 .266)	.014 (-.292 .321)
**Women’s economic rights**	-3.239 (-9.462 2.984)	-2.965 (-8.701 2.770)
**Existence of DV law**	9.288** (.051 18.524)	11.436** (3.250 19.622)
**Quality of DV law**	.178 (-1.498 1.854)	-.234 (-1.808 1.341)
**Unified Democracy Score**	3.168 (-6.112 12.452)	1.122 (-7.566 9.810)
**Women’s political rights**	-10.306 (-25.503 4.890)	-6.468 (-20.758 7.823)
**Seats held by women in national parliament**	.494* (.760 .913)	.454* (.068 .841)
**Political conflict**	-.178 (-2.369 2.014)	-.130 (01.902 2.162)
**Female Literacy rates**	-	-.283** (-49.465–7.065)
**BIC used by Stata**	364.605	359.786
**Adj R squared**	.256	.362

NOTE: The difference between the proportion of women and proportion of men justifying DV in at least one circumstance is the outcome variable; The unstandardised beta coefficients and the 95% CI are presented.

***p < .001

** p < .01

*p < .05;

Based on significance and model fit female primary, secondary and tertiary education, and female labour force participation were not included in final model.

## Discussion

This paper presented country-level gendered analyses of women’s and men’s attitudes to DV across 49 low- and middle-income countries spanning the Caribbean, Central-, South- and South-East Asia, Europe, Latin America and Sub-Saharan Africa. The prevalence rates of DV justification varied widely across countries and geographical regions ranging from 3% in the Dominican Republic to 81% in Timor-Leste. The acceptance of DV was more widespread in Sub-Saharan Africa and South and South-East Asia compared with Latin America, the Caribbean, Central Asia and Europe.

The study found that women are more likely to justify DV than men in 36 of the 49 countries especially in Sub-Saharan Africa and South Asia. This finding is in line with previous studies that focussed on Sub-Saharan Africa [21] and South Asia [18]. The widespread justification of DV by women in Africa and Asia, referred to by Caldwell as the ‘Patriarchal Belt’ [31] can be explained by Kandiyoti’s (1988) theory of ‘patriarchal bargaining’ [32]. According to Kandiyoti many women who live under classic patriarchy are pressured or motivated to conform to the norms of wife blaming in cases of violence as a strategy to mitigate DV [32–34]. Women often internalise the idea that a husband who physically punishes or verbally reprimands his wife has exercised a right that serves her interest. This ‘disciplining’ is seen as a legitimate reprisal for a wife’s disobedience rather than as ‘violence’ [33, 34].

National female literacy rates, a component of a country’s multidimensional poverty index, emerged as the most significant socio-economic protective factor for the acceptance of DV amongst women at the societal level. Levels of justification of DV were lower amongst women in societies with higher levels of female literacy. Female literacy, a measure of women’s empowerment and gender equality status, did not, however, have a significant impact on men’s levels of DV acceptance. This important finding makes a strong case for national policies aimed at improving the status of women and preventing DV to take into account gender disparities in the influence of literacy and education on reducing the societal acceptability of DV and discriminatory gender norms.

This association between poverty and literacy rates is re-iterated by our results that indicate a significant association between a country’s multidimensional deprivation scores and women’s acceptance of DV until we account for national female literacy rates. Severe material, health and education deprivation results in loss in “functioning or freedoms” [35] and alongside rigid gender roles in highly traditional patriarchal societies suggests a strong association between material deprivation and the justification of DV.

The societal acceptance of DV was lower in countries with more gender-equitable economic rights for women that includes, for example, the right to free choice of profession or employment without the need to obtain a husband or male relative’s consent and; the right to gainful employment without the need to obtain a husband or male relative’s consent. These findings suggest that expanding women’s economic rights can serve to challenge existing social norms around gender roles and expectations of women and men. They play an important role in contesting the justification of DV towards a woman who is perceived as transgressing boundaries of wife and/or mother strictly defined for her by her husband and/or male relative and wider society. These rights can thus foster greater agency and transform choices and endowments to achieve desired outcomes [36].

The societal acceptance of DV amongst men was lower in countries with more democratic regimes. Levels of democracy are related to the extent of political participation, the likelihood of a country’s ratification of international treaties and conventions including CEDAW and of having DV legislation [37]. Government structures make laws easier or more difficult to implement. While the existence and quality of DV legislation was associated with lower levels of societal acceptance of DV, this association was not statistically significant. As this is an ecological analysis with a sample size of 49 countries, a focus on statistically significant p values limits our ability to detect very weak effects or draw generalizable conclusions. The existence of an explicit national DV law was, however, associated with a wider gender gap in DV attitudes. Putting laws against DV in place is an important first step for several reasons including defining the different forms of violence against women and signalling government commitment to tackling DV. Policies and laws may result from social movements that, in themselves, may influence societal attitudes. Thus, the temporality and nature of the relationship between enacting laws within the context of government structures and their influence on the societal acceptability of DV is a complex one [38]. Importantly this relationship between the acceptability of DV and DV laws may be impacted on by the quality and comprehensiveness of that legislation with regards to how DV is defined, (in)effective implementation, penalties, evidence required and availability of support services for women. Whilst this is the first analysis to account for the *quality* of the DV law in addition to just its existence, some of the issues around the implementation of national legislation, change in the levels of societal acceptance of DV over time, and their influence on attitudes at the individual- and neighbourhood-level warrant further investigation. These issues are the focus of our forthcoming multilevel analyses papers.

Political conflict significantly increased the likelihood of higher societal acceptance of DV amongst both women and men. The threat women face from sexual violence in conflict areas is increasingly well-documented [39–41]. However, DV, an already acute and pervasive problem, exacerbated during and after armed conflict, has received less attention [42–46]. The tension between ‘traditional’ and ‘modern’ gender roles may be particularly heightened in conflict areas where gender norm shifts are precipitated by financial strains and unemployment resulting from economic downturns [47]–an increased status for women taking on the role of primary earner and men’s perceptions of loss of power and authority [47–48]. Financial dependence, which is more marked in times of conflict, may lead to greater acceptance of DV [49], and wider conflict in the community and society may normalise violence within the household [50]. The findings of the present study contribute to the evidence-base on the societal acceptance of DV in conflict-impacted countries. It emphasises the need to develop strategies to prevent DV by addressing discriminatory gender norms through targeted initiatives in societies affected by political conflict. Our study also highlights the need for more research into understanding the acceptance of DV in conflict-affected societies and the risk and protective factors associated with these DV-justifying attitudes within these regions.

The levels of societal acceptance of DV amongst women was higher in countries with a larger number of women representatives in national parliament. There could be several reasons for these findings being opposite to the hypothesised direction: In countries with high gender inequality, governments may be encouraged, through (inter)national pressure, to ensure greater political participation of women through quota systems for example; elected officials, including women, may reinforce existing social norms including the justification of DV especially in societies where its acceptance is widespread; women’s representation at the national level may also be a crude measure that does not accurately reflect participation and agency at the subnational levels where progress has been slower. The ability of elected officials to influence policies also largely depends on the overall strength of the institutions and committees within which they work. Legislation and administrative procedures that are not women-friendly will limit the impact of female board members [51]. Finally, large and sudden gains in political representation for women are likely to take place when political systems experience broader shifts—after a conflict or political rupture for example [52–53]. This may result in a rapid increase of women entering the political process. For example, Rwanda’s parliament had 56% of women in 2016 compared with just 17% in 1995 [54].

This study suggests that whilst there were some similarities in the drivers of women’s and men’s attitudes towards DV at the aggregate level (women’s economic rights and political conflict), they also differed by gender. Economic factors, poverty and education in particular had a greater influence on the societal acceptance of DV amongst women whilst political and government structures influenced men’s justification of DV. The gender gap in the societal acceptance of DV was significantly predicted by country-level multidimensional poverty, female literacy rates, explicit national legislation on DV and the proportion of seats held by women in national parliament.

### Added value of this study and limitations

There are several noteworthy contributions of this research. This 49-country study is the one of the first to conduct a gendered analysis of the societal acceptance of DV across geographical regions. The number and breadth of countries included in the study gives a more global perspective on violence-justifying attitudes amongst peoples in the global south. It goes beyond existing evidence from multi-country studies that have examined DV attitudes within specific geographical regions, mainly Sub-Saharan Africa [19–21, 24], South Asia [18] and Europe [55], and have largely focussed on individual-level attitudes.

The ecological analyses highlighted the similarities and differences in the prevalence as well as levels and ‘severity’ of DV justification amongst women and men. This study drew on a comprehensive range of diverse high quality internationally comparable metadata to identify the contextual influence of social, economic and political structures on the societal acceptance of DV.

Every effort was made to assess the robustness of the metadata before deciding on the data source for specific country-level predictors. Nonetheless we were constrained by the reliability of existing metadata, some of which relied on national government sources. We addressed this challenge by triangulating data sources and conducting a rigorous scrutiny of coding procedures, decision trees and technical papers preceding decisions to use (sub)-indices. In cases metadata with non-survey year matched data we prioritised metadata preceding the survey year. As changes in contextual factors are slow over time any threat to the validity of the findings was minimised [13].

Given that the aim of this paper was to provide an understanding of the contextual predictors of the societal acceptance of DV, we do not present the multilevel analyses of the independent influence of individual- and contextual-level factors on DV attitudes. We present these hierarchical models in our forthcoming papers. Given the use of cross-sectional data and macro-level analyses we do not make any claims to causation or temporal associations between the predictors and attitudes to DV.

Despite these limitations this study has significant strengths, and provides robust and much-needed evidence on a global scale of the country-level predictors associated with attitudes towards DV. These findings could inform the development of effective prevention intervention programmes and policies on what works at the national level, considering that the economic, social and political interventions would impact on women’s and men’s social norms differently.

### Conclusion

Our study highlighted the widespread acceptance of DV amongst both women and men in low- and middle-income countries with the prevalence and distribution varying widely across countries and geographical regions. In majority of countries in Sub-Saharan Africa and South(east) Asia women were more likely to justify DV whereas this gender difference was reversed in Central/West Asia and Europe. National strategies and public policies aimed at preventing DV and tackling wider gender inequalities might thus need to adopt a geographically differentiated approach to optimally target the societal acceptance of DV.

The evidenced role of structural socio-economic and political empowerment factors on the justification of DV could encourage national governments’, international organisations’ and donors’ to prioritise a multifaceted approach to DV prevention. The study’s findings on the strong impact of political conflict on the societal acceptance of DV suggests the need for the development of prevention interventions tailored to tackling the societal acceptance of DV in these conflict-impacted countries.

Commonly-used dimensions in gender (in)equality indices including women’s labour force participation, number of seats held by women in national parliament and female educational attainment (primary, secondary and tertiary) were not associated with a reduction in societal acceptance of DV. These findings indicate that international DV prevention policies in LMICs need to consider that a sole focus on narrowly defined ‘empowerment’ indicators are insufficient in themselves in influencing and challenging existing discriminatory gender norms.

Finally, our findings also suggest that systemic factors influence women’s and men’s attitudes towards DV differently. This suggests that public health and national DV prevention policies could potentially benefit from a gendered approach to effectively tackle harmful social norms in the global south.

## Supporting information

S1 Table Sample size by country and Demographic and Health Survey year, and prevalence rates of domestic violence against women by an intimate partner within the last 12 months in the sampled countries. (DOCX)

S2 Table Macro-level predictors and data sources. (DOCX)

S3 Table National-level economic, social and political predictors of women’s and men’s attitudes to domestic violence: Multiple linear regression. (DOCX)

## References

[1] Flood M, Pease B. Factors influencing attitudes to violence against women. Trauma Violence Abuse. 2009 4;10(2):125–42. 10.1177/1524838009334131 19383630

[2] Abramsky T, Watts CH, Garcia-Moreno C, Devries K, Kiss L, Ellsberg M, et al What factors are associated with recent intimate partner violence? Findings from the WHO multi-country study on women’s health and domestic violence. BMC Public Health. 2011 2 16;11:109 21324186 10.1186/1471-2458-11-109 PMC3049145

[3] Ali PA, Naylor PB. Intimate partner violence: A narrative review of the feminist, social and ecological explanations for its causation. Aggression and Violent Behavior. 2013 11 1;18(6):611–9.

[4] Jewkes R, Fulu E, Tabassam Naved R, Chirwa E, Dunkle K, Haardörfer R, et al Women’s and men’s reports of past-year prevalence of intimate partner violence and rape and women’s risk factors for intimate partner violence: A multicountry cross-sectional study in Asia and the Pacific. PLOS Medicine. 2017 9;14(9):e1002381 28873087 10.1371/journal.pmed.1002381 PMC5584751

[5] Ackerson LK, Subramanian SV. Domestic violence and chronic malnutrition among women and children in India. American Journal of Epidemiology. 2008 5 15;167(10):1188–96. 10.1093/aje/kwn049 18367471 PMC2789268

[6] Dalal K, Rahman F, Jansson B. Wife abuse in rural Bangladesh. Journal of Biosocial Science. 2009 9;41(5):561–73 10.1017/S0021932009990046 19534836

[7] Fikree FF, Razzak JA, Durocher J. Attitudes of Pakistani men to domestic violence: a study from Karachi, Pakistan. The Journal of Men’s Health & Gender. 2005 3 1;2(1):49–58.

[8] Hindin MJ, Kishor S, Ansara DL. Intimate partner violence among couples in 10 DHS countries: Predictors and health outcomes. 2008 (cited 2017 Nov 27); http://dhsprogram.com/publications/publication-as18-analytical-studies.cfm

[9] Krishnan S, Rocca CH, Hubbard AE, Subbiah K, Edmeades J, Padian NS. “Do Changes in Spousal Employment Status Lead to Domestic Violence? Insights from a Prospective Study in Bangalore, India.” Social Science and Medicine. 2010 1;70(1):136–43. 10.1016/j.socscimed.2009.09.026 19828220 PMC2791993

[10] Koenig MA, Stephenson R, Ahmed S, Jejeebhoy SJ, Campbell J. Individual and Contextual Determinants of Domestic Violence in North India. American Journal of Public Health. 2006 1;96(1):132–138 10.2105/AJPH.2004.050872 16317213 PMC1470450

[11] Nayak MB, Byrne CA, Martin MK, Abraham AG. Attitudes Towards Violence Against Women: A Cross-Nation Study. Sex Roles. 2003 10 1;49(7–8):333–42.

[12] Hindin MJ, Kishor S, Ansara DL. Intimate partner violence among couples in 10 DHS countries: Predictors and health outcomes. 2008 (cited 2017 Nov 27]; http://dhsprogram.com/publications/publication-as18-analytical-studies.cfm

[13] Heise LL, Kotsadam A. Cross-national and multilevel correlates of partner violence: an analysis of data from population-based surveys. Lancet Global Health. 2015 6;3(6):e332–340. 10.1016/S2214-109X(15)00013-3 26001577

[14] Garcia-Moreno C, Jansen HAFM, Ellsberg M, Heise L, Watts CH, WHO Multi-country Study on Women’s Health and Domestic Violence against Women Study Team. Prevalence of intimate partner violence: findings from the WHO multi-country study on women’s health and domestic violence. Lancet. 2006 10 7;368(9543):1260–9. 10.1016/S0140-6736(06)69523-8 17027732

[15] Stöckl H, March L, Pallitto C, Garcia-Moreno C. Intimate partner violence among adolescents and young women: prevalence and associated factors in nine countries: a cross-sectional study. BMC Public Health. 2014 7 25;14:751 10.1186/1471-2458-14-751 25059423 PMC4133076

[16] Walby S, Towers J. Measuring violence to end violence: mainstreaming gender. Journal of Gender-Based Violence. 2017 5 1;1(1):11–31.

[17] What Works to Prevent Partner Violence? An Evidence Overview.—GOV.UK (Internet). (cited 2018 Feb 20). https://www.gov.uk/dfid-research-outputs/what-works-to-prevent-partner-violence-an-evidence-overview.

[18] Rani M, Bonu S. Attitudes Toward Wife Beating A Cross-Country Study in Asia. Journal of Interpersonal Violence. 2009 8 1;24(8):1371–97. 10.1177/0886260508322182 18718881

[19] Uthman OA, Lawoko S, Moradi T. Factors associated with attitudes towards intimate partner violence against women: a comparative analysis of 17 sub-Saharan countries. BMC International Health and Human Rights. 2009;9(1):14. 19619299 10.1186/1472-698X-9-14 PMC2718859

[20] Uthman OA, Moradi T, Lawoko S. Are Individual and Community Acceptance and Witnessing of Intimate Partner Violence Related to Its Occurrence? Multilevel Structural Equation Model. PLOS ONE. 2011 12 14; 6(12):e27738 22194791 10.1371/journal.pone.0027738 PMC3237419

[21] Uthman OA, Lawoko S, Moradi T. Sex disparities in attitudes towards intimate partner violence against women in sub-Saharan Africa: a socio-ecological analysis. BMC Public Health. 2010 4 29;10:223 10.1186/1471-2458-10-223 20429902 PMC2873587

[22] Hayes BE, Boyd KA. Influence of Individual- and National-Level Factors on Attitudes toward Intimate Partner Violence. Sociological Perspectives. 2017 8 1;60(4):685–701.

[23] Tran TD, Nguyen H, Fisher J. Attitudes towards Intimate Partner Violence against Women among Women and Men in 39 Low- and Middle-Income Countries. PLOS ONE (Internet). 2016 11 28;11(11). Available from: https://www.ncbi.nlm.nih.gov/pmc/articles/PMC5125706/10.1371/journal.pone.0167438 PMC5125706 27893861

[24] Uthman OA, Moradi T, Lawoko S. The independent contribution of individual-, neighbourhood-, and country-level socioeconomic position on attitudes towards intimate partner violence against women in sub-Saharan Africa: A multilevel model of direct and moderating effects. Social Science & Medicine. 2009 5 1;68(10):1801–9. 19303687 10.1016/j.socscimed.2009.02.045

[25] Heise LL. Violence against women: an integrated, ecological framework. Violence Against Women. 1998 6;4(3):262–90. 10.1177/1077801298004003002 12296014

[26] Pierotti RS. Increasing Rejection of Intimate Partner Violence: Evidence of Global Cultural Diffusion. American Sociological Review. 2013 4 1;78(2):240–65.

[27] Waltermaurer E. Public justification of intimate partner violence: a review of the literature. Trauma Violence Abuse. 2012 7;13(3):167–75. 10.1177/1524838012447699 22643069

[28] North DC. Institutions. The Journal of Economic Perspectives. 1991;5(1):97–112.

[29] Organisation for Economic Cooperation and Development (OECD): Social Institutions and Gender Index (SIGI). 2012, Accessed on 8-6-2012 http://genderindex.org/country/

[30] Organisation for Economic Cooperation and Development (OECD): Social Institutions and Gender Index (SIGI). 2016, Accessed on 10-8-2017 http://genderindex.org/country/

[31] Caldwell J.C. A Theory of Fertility: From High Plateau to Destabilization. Population and Development Review. 1978; 4: 553–77.

[32] Kandiyoti D. Bargaining with patriarchy. Gender and Society. 1988; 2: 274–290.

[33] Yount K. M., Halim N., Schuler S. R., & Head S. (2013). A Survey Experiment of Women’s Attitudes about Intimate Partner Violence against Women in Rural Bangladesh. Demography, 50(1): 333–357. 10.1007/s13524-012-0143-7 22956416 PMC3716289

[34] Krause KH, Gordon-Roberts R, VanderEnde K, Schuler SR, Yount KM. Why Do Women Justify Violence Against Wives More Often Than Do Men in Vietnam? Journal of Interpersonal Violence. 2016 11 1;31(19):3150–73. 10.1177/0886260515584343 25948647 PMC4636478

[35] Sen A. Development as Freedom. Oxford University Press; 1999 383 p.

[36] United Nations Commission on the Status of Women. 2013. Accessed on 10-09-2014 http://undocs.org/E/2013/27

[37] Giridhar N. 2012 The Global Spread of Domestic Violence Legislation: Causes and Effects. New York University.

[38] Htun M, Weldon SL. When Do Governments Promote Women’s Rights? A Framework for the Comparative Analysis of Sex Equality Policy. Perspectives on Politics. 2010 3; 8(1):207–16.

[39] Johnson K, Asher J, Rosborough S, Raja A, Panjabi R, Beadling C, et al Association of Combatant Status and Sexual Violence With Health and Mental Health Outcomes in Postconflict Liberia. JAMA. 2008 8 13;300(6):676–90. 10.1001/jama.300.6.676 18698066

[40] Kelly JT, Betancourt TS, Mukwege D, Lipton R, Vanrooyen MJ. Experiences of female survivors of sexual violence in eastern Democratic Republic of the Congo: a mixed-methods study. Conflict and Health. 2011 11 2;5:25 10.1186/1752-1505-5-25 22047181 PMC3271036

[41] Peterman A, Palermo T, Bredenkamp C. Estimates and Determinants of Sexual Violence Against Women in the Democratic Republic of Congo. American Journal of Public Health. 2011 6;101(6):1060–7. 10.2105/AJPH.2010.300070 21566049 PMC3093289

[42] Gupta J, Falb KL, Lehmann H, Kpebo D, Xuan Z, Hossain M, et al Gender norms and economic empowerment intervention to reduce intimate partner violence against women in rural Côte d’Ivoire: a randomized controlled pilot study. BMC International Health and Human Rights. 2013;13(1):46. 24176132 10.1186/1472-698X-13-46 PMC3816202

[43] Kohli A, Perrin N, Mpanano RM, Banywesize L, Mirindi AB, Banywesize JH, et al Family and community driven response to intimate partner violence in post-conflict settings. Social Science and Medicine. 2015 12;146:276–84. 10.1016/j.socscimed.2015.10.011 26497097 PMC4643412

[44] Parmar PK, Agrawal P, Goyal R, Scott J, Greenough PG. Need for a gender-sensitive human security framework: results of a quantitative study of human security and sexual violence in Djohong District, Cameroon. Conflict and Health. 2014 5 7;8:6 10.1186/1752-1505-8-6 24829613 PMC4019897

[45] Stark L, Ager A. A Systematic Review of Prevalence Studies of Gender-Based Violence in Complex Emergencies. Trauma Violence Abuse. 2011 7 1;12(3):127–34. 10.1177/1524838011404252 21511685

[46] Tlapek SM. Women’s Status and Intimate Partner Violence in the Democratic Republic of Congo. Journal of Interpersonal Violence. 2015 9 1;30(14):2526–40. 10.1177/0886260514553118 25315479

[47] Horn R, Puffer ES, Roesch E, Lehmann H. Women’s perceptions of effects of war on intimate partner violence and gender roles in two post-conflict West African Countries: consequences and unexpected opportunities. Conflict and Health. 2014 8 4;8:12 25104971 10.1186/1752-1505-8-12 PMC4124472

[48] Hossain M, Zimmerman C, Watts C. Preventing violence against women and girls in conflict. Lancet. 2014 6 14;383(9934):2021–2. 10.1016/S0140-6736(14)60964-8 24923526

[49] Cardoso LF, Gupta J, Shuman S, Cole H, Kpebo D, Falb KL. What Factors Contribute to Intimate Partner Violence Against Women in Urban, Conflict-Affected Settings? Qualitative Findings from Abidjan, Côte d’Ivoire. Journal of Urban Health. 2016 4;93(2):364–78. 10.1007/s11524-016-0029-x 27000124 PMC4835354

[50] Karnofsky E. Familiäre Gewalt und Kindesmissbrauch in Kolumbien. Vol. 4. 200; 37–44.

[51] Falb KL, Annan J, King E, Hopkins J, Kpebo D, Gupta J. Gender norms, poverty and armed conflict in Côte D’Ivoire: engaging men in women’s social and economic empowerment programming. Health Education Research. 2014 12;29(6):1015–27. 10.1093/her/cyu058 25274720 PMC4235567

[52] Kane, M, Oloka-Onyango, J, Tejan Cole, A. Reassessing customary law systems as a vehicle for providing equitable access to justice for the poor. Proceedings of the World Bank’s conference New frontiers of social policy: Development in a globalizing world. 2005 December; Arusha, Tanzania; 2005.

[53] Hassim Shireen. Perverse Consequences? The Impact of Quotas for Women on Democratisation in Africa In Political Representation, ed. Shapiro Ian et al New York: Cambridge University Press 2009; 211–235.

[54] The World Bank. Main report (Internet). The World Bank; 2012 Sep (cited 2016 May 1) p. 1–458. Report No.: 64665. http://documents.worldbank.org/curated/en/492221468136792185/Main-report

[55] Gracia E, Herrero J. Acceptability of domestic violence against women in the European Union: a multilevel analysis. Journal of Epidemiology and Community Health. 2006 2;60(2):123–9. 10.1136/jech.2005.036533 16415260 PMC2588066

