# Supplementary material for: Attitudes towards domestic violence in 49 low- and middle-income countries: A gendered analysis of prevalence and country-level correlates
Source: PLoS One. 2018 Oct 31;13(10):e0206101. doi: 10.1371/journal.pone.0206101 (PMC6209205; doi:10.1371/journal.pone.0206101)
Supplement: S1 Table — (DOCX) [file pone.0206101.s001.docx]

**S1A Table: Sample size by country and Demographic and Health Survey year**

| Country | Survey Year | Women | Men | Total |
| --- | --- | --- | --- | --- |
| Afghanistan | 2014 | 21,520 | 29,461 | 50,981 |
| Albania | 2009 | 6,026 | 7,584 | 13,610 |
| Armenia | 2010 | 3,168 | 5,922 | 9,090 |
| Azerbaijan | 2006 | 5,116 | 8,444 | 13,560 |
| Burkina Faso | 2010 | 14,614 | 17,087 | 31,701 |
| Benin | 2012 | 10,360 | 16,599 | 26,959 |
| Burundi | 2010 | 8,560 | 9,389 | 17,949 |
| Congo DR | 2014 | 17,312 | 18,827 | 36,139 |
| Cote d'Ivoire | 2012 | 5,135 | 10,060 | 15,195 |
| Cameroon | 2011 | 7,191 | 15,426 | 22,617 |
| Dominican Rep | 2013 | 10,306 | 9,372 | 19,678 |
| Ethiopia | 2011 | 14,110 | 16,515 | 30,625 |
| Gabon | 2012 | 5,654 | 8,422 | 14,076 |
| Ghana | 2014 | 4,388 | 9,396 | 13,784 |
| Gambia | 2013 | 3,821 | 10,233 | 14,054 |
| Guinea | 2012 | 3,782 | 9,142 | 12,924 |
| Guyana | 2009 | 3,522 | 4,996 | 8,518 |
| Honduras | 2012 | 7,120 | 22,757 | 29,877 |
| Haiti | 2012 | 9,493 | 14,287 | 23,780 |
| India | 2006 | 74,369 | 124,385 | 198,754 |
| Indonesia | 2012 | 9,306 | 45,607 | 54,913 |
| Kenya | 2014 | 12,819 | 31,079 | 43,898 |
| Cambodia | 2014 | 5,190 | 17,578 | 22,768 |
| Comoros | 2012 | 2,167 | 5,329 | 7,496 |
| Kyrgz Rep | 2012 | 2,413 | 8,208 | 10,621 |
| Liberia | 2013 | 4,118 | 9,239 | 13,357 |
| Lesotho | 2014 | 2,931 | 6,621 | 9,552 |
| Moldova | 2005 | 2,508 | 7,440 | 9,948 |
| Madagascar | 2009 | 8,586 | 17,375 | 25,961 |
| Mali | 2013 | 4,399 | 10,424 | 14,823 |
| Maldives | 2009 | 1,727 | 7,131 | 8,858 |
| Malawi | 2010 | 7,175 | 23,020 | 30,195 |
| Mozambique | 2011 | 8,070 | 13,745 | 21,815 |
| Nigeria | 2013 | 17,359 | 38,948 | 56,307 |
| Niger | 2012 | 3,928 | 11,160 | 15,088 |
| Namibia | 2013 | 4,481 | 10,018 | 14,499 |
| Pakistan | 2013 | 3,134 | 13,558 | 16,692 |
| Rwanda | 2015 | 6,329 | 13,671 | 20,000 |
| Sierra Leone | 2013 | 7,262 | 16,658 | 23,920 |
| Senegal | 2014 | 3,371 | 8,488 | 11,859 |
| SaotaoPrincipe | 2009 | 2,296 | 2,615 | 4,911 |
| Swaziland | 2007 | 4,156 | 4,987 | 9,143 |
| Togo | 2014 | 4,476 | 9,480 | 13,956 |
| Timor Leste | 2010 | 4,076 | 13,137 | 17,213 |
| Tanzania | 2012 | 3,514 | 13,266 | 16,780 |
| Ukraine | 2007 | 3,178 | 13,682 | 16,860 |
| Uganda | 2011 | 2,295 | 8,674 | 10,969 |
| Zambia | 2014 | 14,773 | 16,411 | 31,184 |
| Zimbabwe | 2010 | 7,480 | 9,171 | 16,651 |
| Total |  | 399,084 | 775,024 | 1,174,108 |

**S1B Table: Prevalence rates of domestic violence against women by an intimate partner within the last 12 months in the sampled countries**

| Country | Survey* Year | Emotional violence | Physical violence | Sexual violence | Emotional/Physical/Sexual violence |
| --- | --- | --- | --- | --- | --- |
|  |  |  |  |  |  |
| Afghanistan | 2014 | 34.4 | 45.8 | 6.1 | 51.8 |
| Albania | 2009 | . | . | . | . |
| Armenia | 2010 | . | . | . | . |
| Azerbaijan | 2006 | 5.6 | 9.3 | 1.9 | 11.5 |
| Burkina Faso | 2010 | 7.2 | 8.9 | 1.1 | 12.6 |
| Benin | 2012 | . | . | . | . |
| Burundi | 2010 | . | . | . | . |
| Congo DR | 2014 | 29.4 | 30.3 | 19.9 | 44 |
| Cote d’Ivoire | 2012 | 16.1 | 21.3 | 4.5 | 27.4 |
| Cameroon | 2011 | 32.1 | 28 | 11.2 | 43.2 |
| Dominican Rep | 2013 | 25.6 | 15.1 | 4.4 | 29.1 |
| Ethiopia | 2011 | . | . | . | . |
| Gabon | 2012 | 26.6 | 28.5 | 12 | 39.3 |
| Ghana | 2014 | . | . | . | . |
| Gambia | 2013 | 8.5 | 6.9 | 1.1 | 12.3 |
| Guinea | 2012 | . | . | . | . |
| Guyana | 2009 | . | . | . | . |
| Honduras | 2012 | 20.6 | 10 | 3.3 | 22.5 |
| Haiti | 2012 | 16.7 | 10.4 | 8.6 | 22.1 |
| India | 2006 | 10.8 | 20.5 | 7 | 25.8 |
| Indonesia | 2012 | . | . | . | . |
| Kenya | 2014 | 23.8 | 22.7 | 9.8 | 32.8 |
| Cambodia | 2014 | 17.3 | 9.3 | 3.9 | 19.6 |
| Comoros | 2012 | 6.2 | 4.3 | 1.3 | 8.1 |
| Kyrgz Rep | 2012 | 10.4 | 16.9 | 2.8 | 19.8 |
| Liberia | 2013 | . | . | . | . |
| Lesotho | 2014 | . | . | . | . |
| Moldova | 2005 | 17.1 | 14.1 | 2.5 | 22 |
| Madagascar | 2009 | . | . | . | . |
| Mali | 2013 | 26.2 | 21 | 12.2 | 37.2 |
| Maldives | 2009 | . | . | . | . |
| Malawi | 2010 | 21.2 | 15 | 13.4 | 30.5 |
| Mozambique | 2011 | 29.6 | 25.9 | 6.9 | 39.6 |
| Nigeria | 2013 | 15.3 | 9.3 | 3.7 | 19 |
| Niger | 2012 | . | . | . | . |
| Namibia | 2013 | 21 | 18.7 | 6.6 | 27.9 |
| Pakistan | 2013 | 28.3 | 18 | . | . |
| Rwanda | 2015 | 18.5 | 17.6 | 8.4 | 26.8 |
| Sierra Leone | 2013 | 20.8 | 27.3 | 5.2 | 34 |
| Senegal | 2014 | . | . | . | . |
| SaotaoPrincipe | 2009 | 22.2 | 25.5 | 6.6 | 32 |
| Swaziland | 2007 | . | . | . | . |
| Togo | 2014 | 24.1 | 10.8 | 4.8 | 27.2 |
| Timor Leste | 2010 | 7.4 | 29.6 | 2 | 32.1 |
| Tanzania | 2010 | 30.7 | 32.2 | 13.2 | 42.1 |
| Ukraine | 2007 | 19.3 | 10 | 2.1 | 20.9 |
| Uganda | 2011 | 31 | 24.2 | 19.9 | 42.7 |
| Zambia | 2014 | 17.8 | 21.4 | 13.1 | 31.3 |
| Zimbabwe | 2010 | 22.7 | 20.7 | 13.3 | 35.3 |

***** The domestic violence prevalence rates are matched to the survey year of the countries included in this study. ‘.’ Signifies missing data for the DHS survey year.
