# Supplementary material for: Attitudes towards domestic violence in 49 low- and middle-income countries: A gendered analysis of prevalence and country-level correlates
Source: PLoS One. 2018 Oct 31;13(10):e0206101. doi: 10.1371/journal.pone.0206101 (PMC6209205; doi:10.1371/journal.pone.0206101)
Supplement: S2 Table — (DOCX) [file pone.0206101.s002.docx]

**S2 Table: Macro-level predictors and data sources**

| **Macro-level Predictors** | **Description of variable** | **Data Sources** |
| --- | --- | --- |
| **ECONOMIC AND SOCIAL FACTORS** |  |  |
| Multi-dimensional Deprivation measure | Material deprivation score. Draws on the Bristol method for multidimensional poverty measures in developing countries. It considers nine indicators on housing materials, overcrowding, child malnutrition, health care and education. | Bristol/UNICEF |
| Female Literacy rates | Proportion of literate women in a country | UNESCO UIS and DHS |
| Female Tertiary Education | Ratio of women to men enrolled in tertiary education | UNESCO UIS |
| Women in Secondary Education | Proportion of women completing Secondary Education | DHS |
| Female labour force participation | Proportion of women in the labour force | Women, Business and the Law (WBL)  International Labour Organisation |
| Women’s economic rights | Measure of the degree to which law recognises and governments enforce women’s economic rights | Cingranelli-Richards Human Rights Database |
| GDP PPP per capita (control variable) | GDP per person based on PPP per capita | World Economic Outlook Database (IMF) |
| Working for payment | Percentage of women working for cash or cash and kind | DHS |
| Gini Index | Measure of income inequality | United Nations Development Project |
| Early Marriage <18 | Percentage of women and men married before the age of 18 | UNICEF global databases; DHS |
| **POLITICAL FACTORS** |  |  |
| Women in national parliament | Proportion of women in national Parliaments | Inter-Parliamentary Union; World Bank Gender Statistics database |
| Women’s political rights | Measure of the degree to which law recognises women’s political rights | Cingranelli-Richards  Human Rights Database |
| Unified Democracy Score | Composite scale of democracy estimated using Bayesian techniques also accompanied by estimates of measurement uncertainty. | Developed by James Melton (University College London), Stephen Meserve (Texas Tech University), and Daniel Pemstein (North Dakota State University). |
| Five year conflict mean | Average of total summed magnitudes of all interstate Major Episodes of Political Violence ranging from 1-10 | Centre for Systemic Peace; Armed Conflict Location & Event Data Project |
| **LEGISLATION** |  |  |
| Existence of explicit DV law | Binary measure [ 0=No DV Law; 1= DV law exists] | WBL / WomenStats Database |
| Quality of DV law | Multivariate scale 0=good law and well enforced to 7=weak/no laws and not enforced | WomenStats Database |
| Commitment to CEDAW | 0- Ratified, no reservation, signed Optional Protocol to 3- Did not ratify | WomenStats Database |
| Gender Equality Policy Action Plan | 0-Country has a comprehensive national gender equality action plan to 2- the country has no action plan for gender equality | WomenStats Database |
| Property Rights in law and practice for women | 1- comprehensive law and practice to 4-discriminatory practice and law | WomenStats Database |
