# Supplementary material for: Attitudes towards domestic violence in 49 low- and middle-income countries: A gendered analysis of prevalence and country-level correlates
Source: PLoS One. 2018 Oct 31;13(10):e0206101. doi: 10.1371/journal.pone.0206101 (PMC6209205; doi:10.1371/journal.pone.0206101)
Supplement: S3 Table — (DOCX) [file pone.0206101.s003.docx]

**S3A Table: National-level economic and social predictors of women’s and men’s attitudes to domestic violence: Multiple linear regression**

|  | **Women** | | | **Men** | | |
| --- | --- | --- | --- | --- | --- | --- |
|  | **Model 1** | **Model 2** | **Model 3** | **Model 4** | **Model 5** | **Model 6** |
| **Multidimensional deprivation score** | .234*  [.048 .421] | .215*  [.012 .419] | .065  [-.153 .284] | .071  [-.033 .175] | .074  [-.046 .194] | .036  [-.095 .167] |
| **Inequality Index (Gini)** | -.014  [-.030 .003] | -.013  [-.029 .002] | -.012  [-.027 .003] | -.010*  [-.019 -.001] | -.010*  [-.020 -.001] | -.010*  [-019 -.001] |
| **Women’s economic rights** | -.392*  [-.670 -.094] | -.383**  [-.668 -.098] | -.425**  [-.702 -.148] | -.236**  [-.402 -.070] | -.238*  [-.406 -.069] | -.248**  [-.414 -.082] |
| **GDP PPP** | -27.30e-06  [-83.8 29.3] | -25.9e-06  [-82.9 31.1] | -9.87e-06  [-69.90 45.00] | -5.18e-06  [-38.5 28.2] | -5.38e-06  [-39.00 28.30] | -1.61e-06  [-35.10 31.90] |
| **Early marriage prevalence** | - | .002  [-.012 -.017] | -.009  [-.025 - .007] | - | -.0004  [-.009 .008] | -.003  [-.013 .006] |
| **Female literacy rates** | - | - | -1.834**  [-2.986 -.682] | - | - | -.463  [-1.153 .227] |
|  |  |  |  |  |  |  |
| **BIC used by STATA** | 98.280 | 101.984 | 95.018 | 48.663 | 52.504 | 54.556 |
| **Adj R squared** | .393 | .380 | .496 | .272 | .255 | .265 |

NOTE: The attitudes to DV scale is the outcome variable; The unstandardized beta coefficients and the 95% CI are presented. ***p<.001 ** p<.01 *p<.05; On the basis of significance and model fit female primary, secondary and tertiary education, and female labour force participation were not included in final model.

**S3B Table: National-level political predictors of women’s and men’s attitudes to domestic violence: Multiple linear regression**

|  | **Women** | **Men** |
| --- | --- | --- |
|  | **Model 7** | **Model 8** |
| **Existence and quality of DV legislation** | .065  [-.024 .154] | .018  [-.028 .065] |
| **Unified Democracy Score** | -.497*  [-.975 .020] | -.364**  [-.614 -.113] |
| **Women’s political rights** | -.372  [-1.197 .453] | -.114  [-.547 .319] |
| **Political conflict** | .146**  [.029 .262] | .088**  [.026 .149] |
| **Parliamentary Seats (women)** | .023  [-.000 .046] | .004  [-.084 1.524] |
|  |  |  |
| **BIC used by Stata** | 105.896 | 47.916 |
| **Adj R Squared** | .282 | .393 |

NOTE: The attitudes to DV scale is the outcome variable; The unstandardised beta coefficients and the 95% CI are presented. ***p<.001 ** p<.01 *p<.05

**S3C Table: National-level economic and social predictors of the gender differences in attitudes to domestic violence: Multiple linear regression**

|  | **Model 13** | **Model 14** | **Model 15** |
| --- | --- | --- | --- |
| **Multidimensional deprivation score** | 3.949*  [.240 7.657] | 3.697*  [-.640 8.036] | 1.164  [-3.331 5.659] |
| **Inequality Index (Gini)** | 2.66e-06  [-.323 323] | .006  [-.325 .337] | .022  [-.288 .331] |
| **Women’s economic rights** | -2.659  [-8.578 3.260] | -2.543  [-8.619 3.533] | -3.253  [-8.960 2.453] |
| **GDP PPP** | -.001  [-.002 .001] | -.001  [-.002 .001] | -.029e-06  [-.001 .001] |
| **Early marriage prevalence** | - | -.036  [-.277 .349] | -.164  [-.495 .166] |
| **Female literacy rates** | - | - | -31.014**  [-54.740 -7.288] |
|  |  |  |  |
| **BIC used by Stata** | 379.293 | 383.082 | 379.373 |
| **Adj R squared** | .195 | .177 | .282 |

NOTE: The attitudes to DV scale is the outcome variable; The unstandardised beta coefficients and the 95% CI are presented. ***p<.001 ** p<.01 *p<.05; On the basis of significance and model fit female primary, secondary and tertiary education, and female labour force participation were not included in final model.

**S3D Table: National-level political predictors of gender differences in attitudes to domestic violence: Multiple linear regression**

|  | **Model 16** |
| --- | --- |
| **Existence and Quality of DV law** | .921  [-.860 2.702] |
| **Unified Democracy Score** | -2.691  [-12.224 6.84] |
| **Women’s political rights** | -4.672  [-21.148 11.804] |
| **Political conflict** | 1.001  [-1.324 3.326] |
| **Parliamentary seats (women)** | .430  [-.026 .885] |
| **BIC used by Stata** | 375.371 |
| **Adjusted R squared** | .043 |

NOTE: The attitudes to DV scale is the outcome variable; The unstandardised beta coefficients and the 95% CI are presented. ***p<.001 ** p<.01 *p<.05
